# Challenges in orphan drug development and regulatory policy in China

**DOI:** 10.1186/s13023-017-0568-6

**Published:** 2017-01-18

**Authors:** Alice Cheng, Zhi Xie

**Affiliations:** 1 Rare Genomics Institute, 2657 Annapolis Road, Hanover, MD 21076 USA; 2 State Key Lab of Ophthalmology, Zhongshan Ophthalmic Center, Sun Yat-sen University, 54 Xianlienan Road, Guangzhou, 510040 Guangdong China

**Keywords:** Rare disease, Orphan drugs, Clinical trial, Genetic disease, Chinese medicine, Regulatory policy

## Abstract

While regulatory policy is well defined for orphan drug development in the United States and Europe, rare disease policy in China is still evolving. Many Chinese patients currently pay out of pocket for international treatments that are not yet approved in China. The lack of a clear definition and therefore regulatory approval process for rare diseases has, until now, de-incentivized pharmaceutical companies to pursue rare disease drug development in China. In turn, many grassroots movements have begun to support rare disease patients and facilitate drug discovery through research. Recently, the Chinese FDA set new regulatory guidelines for drugs being developed in China, including an expedited review process for life-saving treatments. In this review, we discuss the effects of these new policy changes on and suggest potential solutions to innovate orphan drug development in China.

## Background

On April 12, 2016, Zexi Wei died from synovial sarcoma, a rare form of cancer. Wei was a 21 year-old college student who had trusted results from the Chinese Internet search giant Baidu to receive a novel treatment for his disease. Later he found that the so-called “novel treatment” was inefficient, and that the hospital was recommended by Baidu based on paid advertising. After his death, it was also revealed that the treatment had proved ineffective in clinical trials in the United States, and that the Stanford team which appeared in the advertisement was not involved at all with the Chinese hospital that administered his treatment [1]. In the weeks that followed, Baidu received public outrage and eventually delisted 2,518 medical institutions and removed 126 million advertisements based on lack of qualifications [2]. While this case study reveals the terrors of the profit-driven healthcare advertising model in China, it also points to a lack of proper rare disease regulatory policy that allowed false medical claims to be propagated.

The Chinese Food and Drug Administration (CFDA) regulates the clinical trials and marketing approval of drugs by evaluating the safety and effectiveness of treatments and ensuring a proper label that highlights the indications as well as the side of effects of drugs. Rare or orphan diseases are very conservatively estimated to affect at least 10 million people in China, and 350 million globally, though the estimates in China may be confounded by limited reported data [3, 4]. Worldwide sales for orphan drugs is projected to be $176 billion by the year 2020, which will comprise almost 20% of total drug sales [5]. Though the patient need is great and the market is huge, the CFDA has yet to design and implement specialized regulation for orphan drug development.

There has been much activity recently with regard to the review and approval system for the CFDA. In November 2015, the definition of a “new drug” was changed to drugs that had never before been marketed *anywhere* in the world, not just in China. Though this is a forward facing policy for encouraging domestic innovation, it may still affect the ability of rare disease patients in China to receive treatment. Currently only 37.8, 24.6 and 52.4% of orphan drugs approved in the US, EU and Japan, respectively, are available in the Chinese market [6]. Thus, this new policy by CFDA may hinder access to life-saving medication for patients without the proper expedited review processes defined for orphan drugs. This paper provides an overview of global orphan drug regulation, discusses financial and scientific challenges in orphan drug development and ends with the current climate and recommendations for orphan drug regulation in China.

## Understanding US FDA and global regulations for orphan drug development

First, an understanding of the United States Food and Drug Administration (US FDA) regulatory process for rare disease clinical trials is necessary for developing regulatory policy in China. This section focuses on the regulatory pathway in the United States because it was the first country to implement a policy for the development of drugs to treat rare diseases with the Orphan Drug Act of 1983, and has since approved the most drugs via this pathway. The US FDA defines an orphan drug as one treating a disease affecting less than 200,000 people in the United States, *or* one that will not be profitable within 7 years following FDA approval [7]. Thus, drugs for economically limiting tropical diseases are also covered. Orphan drugs receive 7 years of market exclusivity beginning after drug approval, which is independent of patient status. Even after this 7-year monopoly expires, new competitors cannot enter the market without proving that their drug is superior to the existing one. Up to one half of research and development costs can be recouped through tax credits, with up to $30 million per year in R&D grants provided for phase I through III clinical trials. These incentives also include a 15-year carry forward provision and 3-year carry back that can be applied once the drug is profitable. In addition, Federal Food, Drug, and Cosmetic Act (FFDCA) Section 526 allows Prescription Drug User Fee Act (PDUFA) user fees to be waives, which results in an average savings of $2 million for companies with less than $50 M in revenue. This provides an incentive for startup companies to develop novel treatments for rare diseases. Section 505A under the FDA Modernization Act of 1997 also grants an additional 6 months of patent exclusivity for drugs that serve the pediatric population, which comprise 50% of the rare disease population [4, 8].

The Orphan Drug Act has almost unanimously been considered successful for advancing rare disease treatment in the US. The financial incentives provided to pharmaceutical companies have increased rare disease research and drug repositioning opportunities. However, it has also created an environment where perverse incentives feedback loops can negatively impact the healthcare system, and ultimately patients. This has most notably been seen in recent news with Martin Shrekli’s strategy of exorbitant price hikes for rare disease drugs [9]. As CEO of Retrophin, Shrekli increased the price of rare disease drug Thiola by 2,000% in 2014. As CEO of Turing Pharmaceuticals, Shrekli executed the same strategy and increased the price of rare disease drug Daraprim by over 5,000%. Valeant Pharmaceuticals CEO Michael Pearson took the same approach, raising drug prices by up to 1,700% over the course of 6 years in order to increase shareholder returns [10]. This was justified to investors as a “capitalistic approach to pricing” based on “what the market will bear.” As the US health insurance system moves toward a model of reimbursement as a percentage (instead of a fixed amount) of a drug’s cost, high prices for rare disease drugs will be passed on to patients, who are then at the mercy of pharmaceutical and shareholder profits. Consequently, some argue that “orphan drug policies have the paradoxical effect of creating new orphan patients! [11]”.

Though the US has pioneered structure in rare disease research and policy, differences in regulatory policy exist for countries in Europe and Asia (Table 1). One important discrepancy among regulatory policies is the lack of a global definition for what constitutes a rare disease. Therefore, drugs considered for a rare indication in Europe may not be approved in the United States under an expedited pathway.

**Table 1:** Comparison of orphan drug regulation across the US, Europe, Japan, Korea, Taiwan and China

Country	Rare disease definition	Regulatory agency	Market exclusivity	R&D/Tax credits	Approval time	Approved orphan drugs
United States [43–46]	<200,000 (0.1% of population)	Food and Drug Administration (FDA)	7 years	50% tax credit for clinical studies	6 months	569
China [34]	Suggested prevalence 300,000–500,000	China Food and Drug Administration (CFDA)	N/A	N/A	N/A	N/A
European Union [27, 43, 47–49]	<215,000 (0.05% of population)	European Medicines Agency (EMA)	10 years	Yes, country dependent	5 months	116
Japan [16, 43]	<50,000, maximum 0.05% of population or no available treatment	Pharmaceuticals and Medical Devices Agency (PMDA)	Up to 10 years	Waived consultation fee ($20 K USD), up to 50% of development costs, 12% tax exemptions, 14$ corporate tax, ~25% reduction in review fees, portion of profits exceeding 100 M yen returned to government	10 months (vs 12 months for regular drugs)	203
Korea [16]	<20,000 or no available treatment, less than $5B won production costs/import	Korea Food and Drug Administration (KFDA)	6 years	50% subsidized application fee	6–9 months	184
Taiwan [16, 27, 43]	<2300 or 0.01% of population	Taiwan Food and Drug Administration (TFDA), Center for Drug Evaluation (CDE)	10 years	Financial subsidies not disclosed	6–10 months	77 drugs, 40 special nutrients

## Financial considerations for orphan drug development

However, most orphan drugs are still unaffordable in China. With a 5% patient co-pay, only three generic orphan drugs were considered affordable by middle-income Chinese patients [6]. Over 100 commercial health insurance companies exist in China for those who are able to afford an additional policy to supplement government insurance [12]. However, chronic rare diseases are not well defined for coverage, with approximately 10–15 rare diseases that are covered. A ceiling coverage of only 100,000 yuan is provided for chronic disease in one plan by Taikang Life Insurance Co., Ltd. For families with children suffering from a rare disease, a financial contribution can easily surpass 40%, causing a catastrophic financial burden. In addition, many international drugs not yet approved by the CFDA are sold in the “gray market” to patients, who pay cash out of pocket. Many patients cannot afford these treatments and forego them altogether. Though CFDA incentivizes companies to develop orphan drugs, patients must still be able to afford these treatments in order for the market to be sustainable. One proposal by Professor Longjun Gu of Shanghai JiaoTong University advocates for a rare disease specific fund composed of contributions from the national medical insurance, commercial insurance and government charity [13]. This would help cover costs that are beyond what patients are able to pay.

Patients have also suggested ways to counter this with government intervention; FDA could expedite approval of generics or competing rare disease drugs once the price of a drug is increased past a certain amount [10]. Japan includes this in its rare disease policy, requiring companies receiving profits in excess of 100 M yen for rare disease drugs to pay back a portion of those profits to the government (Table 1). The National Institute for Health and Clinical Excellence (NICE) of England and Wales defines each quality-adjusted life-year as £20,000–30,000, with drugs possessing a quality-adjusted life-year (QALY) of less than £20,000 being more likely to receive reimbursement [14]. However, metrics for reimbursement are often not disclosed or clearly defined in other countries. Additionally, it is difficult to use these traditional economic analyses to evaluate orphan drugs, which are used to treat such a small subset of the population. Modified QALY analyses have been proposed, including a person trade-off (PTO) approach to determine how many treated individuals would be equivalent to one healthy individual [15].

Current provincial and city initiatives provide medical insurance and drug reimbursement for rare disease patients. In Shanghai province, standard medical coverage of up to 200,000 yuan is provided for 12 specific rare diseases, though this falls short of an estimated 2 million yuan annually needed per patient to cover rare disease treatment [16, 17]. Since 2012, the city of Qingdao in the Shandong province has covered treatments fees of up to 400,000 yuan for all, including orphan, diseases. Though these initiatives help relieve financial burdens of some rare diseases, a national program with guaranteed coverage for clearly identified rare diseases will democratize healthcare for all rare disease patients in China.

For drugs that are currently approved in other countries, Chinese FDA should lay out specific regulations for approval or sale. Simplifying registration of foreign-approved drugs will prevent redundancy in regulation and economic burden, and increase availability of treatment for patients. Regardless of approach, political and economic considerations will factor into geographic-specific regulations. As validation for government subsidies, patient registries with access to de-identified data have been proposed to advance public health [18]. For ultra-orphan drugs, a direct distribution model that trims costs from distributors also has potential to carry savings directly to patients [19].

## Scientific and technical considerations in research and clinical trials

**Fig. 1:**
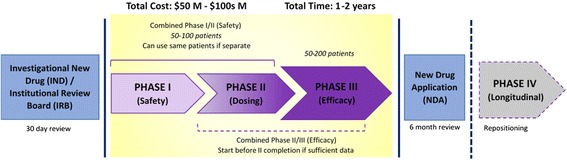
Regulatory schematic of orphan drug development in the United States

A major challenge in the approval of rare and ultra-rare orphan drugs is clinical trial design. Typically, clinical trial phases 1, 2 and 3 are conducted linearly, with each phase completed and evaluated in order to inform design of the next phase (Fig. 1). However, new seamless designs for clinical trials are being implemented, with statistical analyses that allow the next phase to begin before the primary phase has completed [20, 21]. These innovative new trial designs require enough statistical power and can be quite useful for expediting time-to-market as well as reducing the number of enrolled patients, saving the sponsor costs. These options are currently being evaluated for rare disease trials, which can be even more challenging due to the small patient population [22]. One recently approved drug for the treatment of hereditary orotic aciduria, a disease that is only known to affect 20 people worldwide, completed a clinical trial with only four patients over 6 weeks [23].

Patient recruitment and retention are the biggest challenges in clinical trials. The Clinical Trial Act legislation was implemented in the US to regulate patient compensation during clinical trials. Patients are able to receive up to $2,000 USD for participating in clinical trials without counting toward taxable income. A standard compensation per visit versus per trial, for traveling and lodging costs or for home monitoring, is set by the sponsoring pharmaceutical company instead of the clinical research organization (CRO), and can vary across trials. Whereas average dropout rates across typical clinical studies can still exceed 30%, this number may be much lower for rare disease trials [24]. A new study shows that rare disease patients are more likely to accept risks in trying new medications, with drug response ranking as the most important outcome regardless of treatment modality [25]. In addition, 45% of patient respondents from a survey taken by the National Organization for Rare Disorders (NORD) in 2014 expressed that they were willing to use experimental treatments [19]. These data indicate that rare disease patients are easier to recruit for and retain during a clinical trial. This reduces redundancy in patient recruitment costs, where many trials must over-recruit to compensate for drop-out rates.

In a draft guidance to industry on common issues in orphan drug development by the US FDA, natural history and disease pathophysiology are cited as important for having a foundational understanding of the disease in order to develop appropriate study endpoints [26]. This is especially important for rare disease trials, which often include pediatric patients with life-threatening conditions. In the US, the Office of Orphan Products Development specializes in researching and implementing orphan drug regulation. A specialized office for orphan diseases does not yet exist within the CFDA, but will be necessary as orphan drug and personalized medicines become a major focus in global healthcare [27].

Without rigorous clinical trial design and reporting requirements, it will be difficult to evaluate the safety and effectiveness of drugs. An unregulated landscape can be seen in traditional Chinese medicine (TCM) clinical trials, where over 60% of studies did not adhere to the standard Consolidated Standard of Reporting Trials requirements [28]. Training on proper record keeping and informed consent processes must therefore be rigorous and strictly implemented in order for Chinese hospitals and CROs to be competitive for domestic and international drug development.

A cultural component should also be considered. Though many traditional Chinese medicines (TCMs) are merely theatrical placebos, the market is still booming for herbal cures [29]. The perceived success and desperation of patients for a cure contribute to the placebo response not only in China, but also in the United States. Though the placebo effect was originally higher in China than in the US, the US has started to see increasing placebo responses over the past 23 years that now surpass the drug-placebo effectiveness of other countries [30]. This effect could be exacerbated by drug pricing, with studies showing that the higher the price of the drug, the higher the placebo response [31]. It may be fair to say that this effect could be exaggerated in China, where paranoia of domestic manufacturing quality lead to increased belief in foreign treatments. The Chinese “anti-placebo” concept has been documented as far back as 1993, when presumably ill-fated Chinese-Americans died up to 5 years sooner than Caucasian patients from a variety of causes simply because of their belief in a negative astrological sign [32]. Alternatively, this phenomenon may also negatively impact patients who have an increased willingness to participate in a study without the proper informed consent process, such as was the case with Mr. Wei and his family. Therefore, educational materials and the patient consent process should be clearly outlined for all studies.

## Rare disease initiatives in China

In the past, rare diseases did not receive much attention in China. This could partly be attributed to lack of awareness, and partly to limitations in diagnostic technologies. A genetic skeletal disease study from 2012 indicates that genetic testing was performed for only 1.16% of over 16,000 patients [33]. With growing awareness and technological capabilities, China is working toward a more standardized and comprehensive rare disease ecosystem. Recent changes proposed by the CFDA include expedited review for rare disease drugs; however, this pathway is not well defined. Although there is no official definition for a rare disease in China, a bottom-up analysis of cost-effectiveness proposes that rare diseases be defined as affecting between 300,000 to 500,000 of the population [34]. This is based on a $1.2 billion drug development cost and an optimistic insurance reimbursement ceiling of $50,000 USD per year per patient.

In 2013, the China Rare Diseases Prevention and Treatment Alliance launched a national pilot to advance rare disease healthcare, involving over 100 medical centers over 13 provinces and 0.7 billion people [35]. The project focuses on 20 rare diseases and hopes to use outcomes to develop an executable plan for improving rare disease healthcare by 2018, with the following three aims:

1. Develop and pilot clinical guidelines and standards for the 20 specified rare diseases. Optimized guidelines after pilot testing will be implemented in hospitals nationwide.
2. Establish a patient registry based on retrospective medical records dating back to 2003 along with newly diagnosed cases. All data will be de-identified using Common Data Elements (CDE). A public patient registry will allow advocacy organizations to register their own patients.
3. Implement nine single gene and seven next-generation sequencing analyses for 15 rare diseases to encourage molecular genetic testing analyses in rare disease diagnosis, research and care.

In Europe, an additional “ultra-orphan” designation has been proposed for diseases affecting less than 1 in 50,000 of the population (compared to affecting 1 in 2,000 for rare diseases) [36]. In the United States, ultra-rare diseases are generally defined as affecting less than 20,000 people [19]. This designation was also officially proposed via the HR3737 ULTRA (Unlocking Lifesaving Treatments for Rare-Diseases Act) in December 2011 to approve certain orphan drugs based on surrogate endpoints [37]. A report on cost-effectiveness of ultra-orphan drug development in Europe concluded that traditional criteria for cost-effectiveness cannot be used for ultra-orphan drugs with such small patient populations [36]. Thus, challenges remain for justifying development costs and subsidies of drugs for ultra-rare diseases. While it may be premature to designate an “ultra” for currently undefined “rare” diseases in China, this points to a need for new ways of evaluating the economic impact of orphan drug development. As health insurance becomes more of a focal point for the rapidly aging population, this will also play into the broader scope of drug reimbursement.

Broader government initiatives for fostering rare disease research include hosting national scientific forums, encouraging the formation of research consortiums and providing an avenue for publication. The Intractable and Rare Diseases Research journal was established in 2013 with support from the Japan Society for the Promotion of Science, the Ministry of Education, Culture, Sports, Science and Technology and the Japanese government [38]. China could greatly benefit from an English language publication platform to advance rare disease research, which will also enhance China’s global scientific competitiveness.

## Collaborating with patient advocacy groups to build registries and raise awareness

The purpose of drug regulation is to make sure safe and effective treatments can be developed, and to help provide a balance in the market while advancing public health. Therefore, most regulation by FDA or CFDA is developed by scientific and regulatory experts. Regulatory agencies must work closely with patient advocacy groups to develop policies that can be feasibly implemented. While the government has the power to implement and enforce rare disease policies, it is ultimately the patient community that drives change. In the US, NORD is credited for instituting the Orphan Drug Act of 1983.

In fact, patient advocacy organizations are not only responsible for changes in legislation, but are also focused on research. Ninety percent of rare disease patient organizations have a goal of advancing research, with 95% participating in at least one research-related activity in the past year [39]. Fifteen percent of these organizations identified research as their primary goal. The Chinese Organization for Rare Disorders (CORD) was established in 2013 and is the prominent patient advocacy organization for rare disease patients. This organization hosts 23 member organizations and serves as a means of information dissemination and a voice for patients to government and industry [40]. Ongoing outreach campaigns have educated over 100 million people in China about rare diseases. Other public institutions such as the Shandong Academy of Medical Sciences, Tsinghua University, the Chinese Charity Foundation and the China Health Education Center have also supported programs to spread awareness and worked to advance rare disease research. These organizations will be the biggest advocates and allies in the CFDA’s implementation of orphan drug regulation.

While rare disease patients are generally informed about their disease, they may not be aware of clinical trial opportunities. Additionally, patients may not trust for-profit companies that reach out to participate in trials. Working with patient advocacy organizations helps build trust with patients and may increase retention during the study. Similar to the generic drug marketing campaign, the CFDA can work with patient advocacy groups in China to inform patients about clinical trials. A national registry with standardized clincal trial demographic and patient information can also inform clinical trial site selection. Building a national health registry for patients with rare diseases in China can be done under government guidance, with participation between hospitals, research institutions and patient advocacy groups [41].

## Conclusions

This paper extensively compared orphan drug development and regulatory policy in China and the US. With many political, economic and cultural differences, China cannot just base its regulations directly on the US model. In contrast to the mostly private healthcare system in the US, where registries can be fragmented across patient advocacy groups and hospitals, a healthcare system that is mostly public in China should take advantage of available data to create aggregated databases for diseases and genomic information. Even without a registry, existing software tools such as DISMOD II can be used to aggregate patient data and analyze rare disease epidemiology across participating hospitals [42]. We continue to advocate for the five suggestions proposed by the National People’s Congress and Chinese People’s Political Consultative Conference of 2009: 1) establishing a definition for rare diseases, 2) developing an orphan drug reimbursement system, 3) proposing a clear and simple approval pathway for imported orphan drugs, 4) promoting rare disease research through policy, and 5) developing government-supported programs for rare disease patients [17]. The Chinese FDA should also continue working with patient advocacy groups and research institutions to identify the most prevalent diseases and patient needs. Clearly defining regulatory policy for orphan drugs will benefit both patients and research organizations, and allow China to serve as a global player in rare diseases.
